# Recurrent Plasmacytomas after Allografting in a Patient with Multiple Myeloma

**DOI:** 10.1155/2012/168785

**Published:** 2012-12-25

**Authors:** Allen N. Stawis, Diane Maennle, Moreno Festuccia, Zia Uddin, Benedetto Bruno

**Affiliations:** ^1^Department of Hematology and Oncology, Webber Cancer Center, St. John Macomb-Oakland Hospital, Warren, MI 48093, USA; ^2^Department of Clinical Pathology, St. John Macomb-Oakland Hospital, Warren, MI 48093, USA; ^3^Division of Hematology, University of Torino, Via Genova 3, 10126 Torino, Italy

## Abstract

Extramedullary recurrence in multiple myeloma patients has been reported after both autologous and allogeneic hematopoietic cell transplantation and, more recently, after treatment with so-called new drugs with potent antimyeloma activity. Only a very few sizable reports focused on its clinical presentation and its incidence, which may be highly underestimated, and most observations are based on single patients reported from several institutions. Given the unusual sites of recurrence, diagnosis may be rather difficult and delayed treatment may play a relevant role in prognosis. Here we report a case of a myeloma patient, initially treated with an allograft, who enjoyed an eleven-year disease-free remission with very good quality of life. She eventually relapsed first with an extramedullary plasmacytoma in the breast and, two years later, with a right atrial cardiac mass.

## 1. Introduction

Allografting is considered a potentially curative treatment for multiple myeloma, although long-term disease control remains an issue [1]. The frequency of posttransplant extramedullary recurrence and of extramedullary plasmacytoma (EMP) without marrow plasma cell infiltration is reported variably among institutions even after both autologous and allogeneic transplantation [2–11]. Similarly, the selection of diagnostic laboratory tests and imaging techniques vary among physicians, although early detection of recurrence may have a significant impact on treatment outcome. Here we report a case of EMP in the breast and in the atrium occurring 11 and 13 years after an allograft, respectively. We also illustrate the potential usefulness of serum free light chains (FLC) and their ratio in the diagnosis and followup of EMP.

## 2. Case Presentation

In June 1998, a 45-year-old lady complained to her family physician about fatigue, pain in the lower extremities, and inability to concentrate at her job. The physical examination revealed the patient to be pale with a hemoglobin of 4.9 g/dL. She was then referred to a hematologist-oncologist. Immunofixation (IFE) studies of serum and urine were positive for monoclonal serum proteins (M-proteins). Bone marrow biopsy submitted in formalin revealed 100% involvement by monoclonal plasma cells. No cytogenetic or FISH studies were routinely performed at that time. A metastatic bone survey showed no osseous abnormality. She was staged as Durie-Salmon Stage II, International Staging System II. Induction therapy consisted of 2 cycles of standard VAD (vincristine, adriamycin, and dexamethasone) regimen. After the first cycle, the serum and urine remained positive for M-proteins. A second cycle was given and there was no adequate response. Given the unsatisfactory response and the young patient age, she underwent an allograft from her sister in December 1998, after a conditioning with total marrow irradiation, busulfan and cyclophosphamide. The posttransplant course was rather uneventful except for a hepatitis B reactivation syndrome (after immunosuppressant therapy) successfully treated with lamivudine. Two years after the allograft, the bone marrow evaluation showed complete remission with less than 1% polyclonal plasma cells. Monoclonal proteins were not detected in serum or urine by IFE, and the 24 hour urine total protein was too low for quantification (Table 1).

The patient continued to do well and pursued a full-time job during the eleven year interval after the allograft. She remained free of myeloma (normal bone marrow, no evidence of discrete lytic or blastic lesions, negative for monoclonal proteins in serum and urine from IFE studies, and urine total protein too low for quantification) until she complained about a right breast mass located towards the axilla during a follow-up exam in 2009. Her latest mammogram in 2001 had been negative. The 2.1 cm mass consisted of sheets of monoclonal plasma cells and was diagnosed as solitary plasmacytoma. The EMP was excised and radiation therapy was delivered for five weeks to the right breast tumor bed at a total dose of 5040 cGY. At this time, the bone marrow examination revealed no increase in plasma cells (<1%), and the CT scan indicated no evidence of metastatic disease to the chest, abdomen, pelvis, or lytic or blastic lesions within the skeleton. Serum and urine were negative for M-proteins (IFE studies), and the protein in urine was too low for quantification. For the following two years the patient remained free of any obvious clinical problem and was able to work full-time. Bone marrow examinations in 1999, 2000, and 2009 did not show myeloma recurrence.

**Figure 1:**
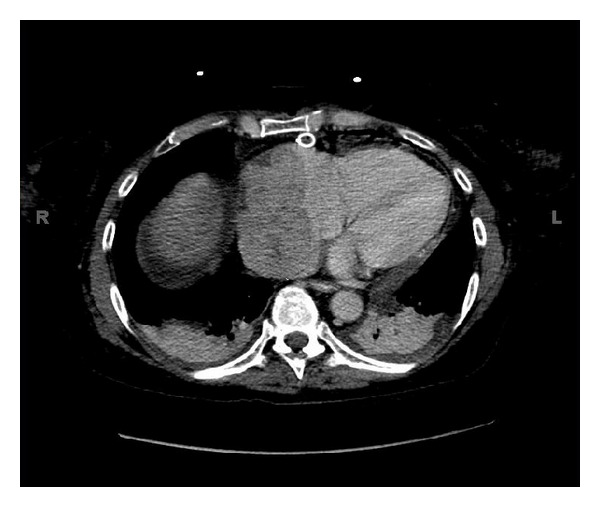
Right cardiac atrial mass shown by CT scan extending into the inferior vena cava, involving the superior vena cava as it enters the atrium, and pericardial effusion.

Thirteen years after the allograft, she complained of shortness of breath and dyspnea on exertion. She denied any chest pain and any palpitations. The patient was put on albuterol, but continued to have shortness of breath. An echocardiogram in her family physician's office indicated the presence of a “large right atrial thrombus”, and she was immediately referred to the hospital emergency department. A venous ultrasound of the right and left lower extremities showed no evidence of venous thrombosis. Imaging studies ruled out pneumothorax and/or pulmonary embolism. Echocardiogram documented a normal ejection fraction (60–65%); however, a mass measuring 6.7 × 3.3 cm was seen in the right atrium encroaching the right atrial wall and a large pericardial effusion with tamponade. CT confirmed a large right cardiac atrial mass, extending into the inferior vena cava and also involving the superior vena cava as it enters the atrium and the pericardial effusion (Figure 1). The patient then underwent a pericardial window to drain the pericardial effusion. The effusion cytology showed that 50% of the cells were plasma cells, which was confirmed by immunoperoxidase CD138 positive staining. In situ hybridization stains for kappa and lambda chains indicated that almost all of the plasma cells were positive for lambda light chains. The diagnosis of monoclonal plasmacytosis was made. Biopsy of the pericardial tissue ruled out other malignancies, amyloidosis, and vasculitis.

**Figure 2:**
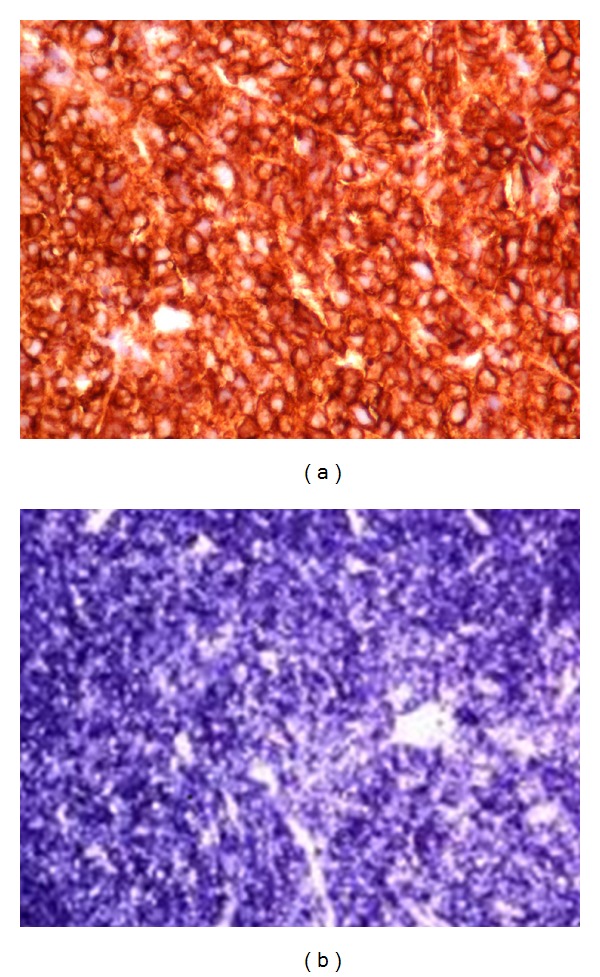
(a) Diagnosis of extramedullary plasmacytoma confirmed by immunoperoxidase staining for CD138 and (b) in situ hybridization for lambda chains which confirmed plasma cell monoclonality.

Transcatheter biopsy of the right atrial cardiac mass by fluoroscopic guided road-mapping was performed. The tissue sample consisted of sheets of plasma cells. The diagnosis of EMP was confirmed with a combination of immunoperoxidase staining for CD138, which showed strong positivity, and in situ hybridization for kappa and lambda, which confirmed monoclonality since all of the cells stained for lambda chains (Figures 2(a) and 2(b)). Flow cytometry was negative due to poor cell survival. Bone marrow biopsy showed normocellular marrow with 100% donor chimerism. The B cells were polyclonal by flow cytometry and there was no evidence of monoclonality within the plasma cells. In situ hybridization revealed a normal kappa/lambda ratio of 1.4 : 1. MRI of the spine was unremarkable, without any evidence of canal or neural foraminal stenosis or of myelomatous disease. Chemotherapy with monthly cycles of bortezomib and dexamethasone was initiated and the patient was monitored with serial serum FLC assay, CT and echocardiography. After four months, the tumor exhibited a substantial reduction in size measuring 4.7 cm on echocardiogram and CT scan. Serum free lambda chain concentration decreased linearly, with simultaneous increase in the kappa/lambda ratio (Table 2). The patient is alive and well at this writing, in partial remission by the International Uniform Response Criteria for multiple myeloma, 14 years posttransplant [12–14]. She is receiving continuous chemotherapy (bortezomib, dexamethasone, and now Cytoxan) despite an episode of DVT in the right leg (3/2012) and a recent syncopal event (7/2012). The atrial mass measures 4.1 × 0.9 cm in greatest dimensions by transesophageal echocardiography (11/2012).

## 3. Discussion

We believe that this case of a lambda chain myeloma patient, who had an allograft, experienced 2 EMP, after a long clinical remission, exhibits rather interesting features: (a) a long disease-free survival without maintenance/consolidation treatment with very good quality of life after the allograft; (b) extramedullary relapse as solitary EMP of the breast 11 years after the allograft; (c) two years later a second EMP of the right cardiac atrium. Bone marrow studies invariably showed no evidence of marrow plasma cell infiltration with 100% donor chimerism, thus indicating the confinement of myeloma relapse only to the soft tissues.

Extra-medullary relapse of myeloma with sparing of the marrow after both autologous and allogeneic transplantations and, more recently, after therapy with novel agents such as thalidomide, lenalidomide, and bortezomib, is a mode of recurrence the frequency of which is undetermined and may be highly underestimated. Only a few reports have evaluated EMP incidence, clinical characteristics, and prognosis, and most of these are summarised in case reports [2–11]. Moreover, definitions of EMP lesions vary among study groups [15].

Varettoni et al. reported an incidence of extramedullary recurrence of 6% on a series of 1003 myeloma patients, which was found to be associated with shorter overall and progression survivals [9]. Zeiser et al. reported a higher incidence after autologous and allogeneic transplantation [8]. Numerous sites of involvement such as lungs, soft tissues, pericardium, skin and central nervous system were reported. The mechanisms underlying the selective involvement of extramedullary sites remain undefined. It may be postulated that these mechanisms may be due to recurrence in sanctuary sites where myeloma cells are resistant to intense therapy, or to a stronger putative graft-versus-myeloma effect in the marrow as compared with peripheral tissues after an allograft.

A major concern for clinicians is the early detection of the recurrence, because delayed diagnosis may compromise treatment efficacy. Delayed diagnosis is particularly frequent when the clinical presentation of the recurrence is rather unusual, skeletal radiological surveys are negative, and monoclonal paraproteins are undetectable. Even though techniques such as MRI and PET imaging may be of value for monitoring the patient, more readily available and less expensive laboratory assays should be sought. In our patient, serum (SPE) and urine protein electrophoreses (UPE), and IFE (serum and urine) were negative at the time both breast and atrial masses were noted, therefore indicating the lack of sensitivity of these tests for the early diagnosis of solitary EMP. By contrast, only the serum free lambda chains were ten fold higher than the normal value, and the free light chain ratio was abnormal, which suggests its potential usefulness in the early detection of posttransplant extramedullary recurrence.

Although the role of serum FLC assay is still a matter of debate to determine stringent clinical complete remission, it may be particularly relevant for early detection and followup of EMP where more sensitive methods such as flow cytometry and/or FISH analysis cannot be performed on an uninvolved bone marrow [16–18]. In addition to the light chain myeloma, 95% of the intact monoclonal immunoglobulin (M-Ig) myelomas have an abnormal serum FLC concentration and ratio. The malignant plasma cells most often screte FLC in addition to intact immunoglobulins. Recently in both nonintensive or more intensive therapy in M-Ig MM, three new developments were reported: (1) relapse as EMP with a marked increase in serum FLC (also designated as light chain escape) in the absence of a parallel rise in M-Ig [19]; (2) clonal change at relapse, identified as patients who produced a M-Ig at presentation but relapsed with rising production of serum FLC and stable or falling M-Ig concentration in the serum [20, 21]; (3) early detection of relapse of M-IgA MM after autologous SCT by newly developed Hevylite assay [22], which utilizes antibodies specific for the intersection of the heavy chain and light chain of each immunoglobulin, and allows separate quantification of each immunoglobulin heavy and light chain combination [23]. Overall, reports suggested that FLC were more sensitive for the early detection of EMP as compared to other laboratory tests. We now incorporate the following protocol in the clinical pathway for monitoring myeloma patients in clinical remission after transplant: (i) serum FLC assay prior to the start of treatment; (ii) serum FLC assays every two weeks for three months to evaluate response to treatment; (iii) serum FLC assays every three months unless the patient develops overt myeloma symptoms. A prospective study on a large cohort of myeloma patients is in progress. 

EMP and extramedullary recurrence are rather challenging to treat. A “standard” therapeutic approach is not currently available. At present, most treatments are individualized and include a wide range of strategies such as radiation therapy, new drugs, and donor lymphocyte infusions after an allograft [2–11]. In our patient, the first EMP in the breast was treated with surgical excision and radiotherapy that allowed for a remission duration of 2 years until a new mass in the right cardiac atrium led to the diagnosis of a second EMP which responded to bortezomib and dexametasone.

Current large prospective clinical trails should include side studies that longitudinally evaluate incidence, clinical presentation, and prognosis of extramedullary recurrence. Moreover, retrospective analyses on large series of patients from study groups such as the European Myeloma Network and/or Bone Marrow Transplantation Registries may be helpful to better define this clinical entity. Finally, simple and sensitive diagnostic tests are needed for timely diagnosis that may allow for improved treatment outcomes.

## Figures and Tables

**Table 1:** Summary of laboratory tests^a,b^.

Date	Hb^c^	Ca	BUN	Cr	BMG	IF-Serum^c^	IF-Urine^d^	Remarks
June 1998 Diagnosis	**4.9**	9.1	10	0.7	**3.9**	*Monoclonalλ*	*Monoclonalλ*	Urine protein = **17.9** g/24 hrs, **85% ** *freeλchains *
December 1998								
*Allogeneic Stem Cell Transplantation *								

December 1999	11.8	9.4	11	0.6	2.1	*Normal *	*Normal *	Urine protein = 0.2 g/24 hrs

December 2000	14.0	9.6	13	0.7	1.3	*Normal *	*Normal *	*Urine protein too low for quantification *

December 2001 *Mammogram: No evidence of malignancy *	14.0	10	14	0.6	1.3	*Normal *	*Normal *	*Urine protein too low for quantification *

June 2009 *Right breast plasmacytoma (*λ*): surgery plus radiation *	14.0	10.3	17	0.6	1.6	*Normal *	*Normal *	*Urine protein too low for quantification *

July 2011 *Right atrium plasmacytoma (*λ*) *	14.1	9.8	14	0.6		*Normal *	*Normal *	**17** mg/dL urinary protein (random)

October 2012	13.8	9.9	16	0.7	n.d.	n.d.	n.d.	*Urine protein too low for quantification *

^a^Serum reference values (abbreviations): hemoglobin (Hb) = 12–15 g/dL, calcium (CA) = 8.2–10.2 mg/dL, urea (BUN) = 7–22 mg/dL, creatinine (Cr) = 0.2–1.3 mg/dL, beta-2 microglobulin (BMG) = 0.7–3.4 mg/dL, ^b^urine reference values: 24 hours rotein = less than 0.15 g/24 hrs, random protein = not established, ^c^IF-Serum: serum immunofixation, ^d^IF-urine: urine immunofixation; n.d.: not done.

**Table 2:** Summary of serum free light chains assays.

	Free Kappa (mg/dL) Normal: 0.33–1.94	Free Lambda (mg/dL) Normal: 0.57–2.63	Free Kappa/Lambda Ratio Normal: 0.26–1.65	Chemotherapy with bortezomib and dexametasone
August 2011	0.78	**27.7**	**0.03**	Start Cycle 1
September 2011	0.18	**3.3**	**0.05**	Start Cycle 2
	0.15	**3.2**	**0.05**	
	0.24	**2.8**	**0.09**	
October 2011	0.98	2.4	**0.41**	Start Cycle 3
November 2011	0.87	1.3	0.66	Start Cycle 4
October 2012	0.75	1.2.	0.62	

## References

[1] Bruno B, Giaccone L, Sorasio R, Boccadoro M (2009). Role of allogeneic stem cell transplantation in multiple myeloma. *Seminars in Hematology*.

[2] Wirk B, Wingard JR, Moreb JS Extramedullary disease in plasma cell myeloma: the iceberg phenomenon.

[3] Madan S, Kumar S (2009). Review: extramedullary disease in multiple myeloma. *Clinical Advances in Hematology and Oncology*.

[4] Pérez-Simón JA, Sureda A, Fernández-Aviles F (2006). Reduced-intensity conditioning allogeneic transplantation is associated with a high incidence of extramedullary relapses in multiple myeloma patients. *Leukemia*.

[5] Terpos E, Rezvani K, Basu S (2005). Plasmacytoma relapses in the absence of systemic progression post-high-dose therapy for multiple myeloma. *European Journal of Haematology*.

[6] Byrne JL, Fairbairn J, Davy B, Carter IG, Bessell EM, Russell NH (2003). Allogeneic transplantation for multiple myeloma: late relapse may occur as localised lytic lesion/plasmacytoma despite ongoing molecular remission. *Bone Marrow Transplantation*.

[7] Alegre A, Granda A, Martínez-Chamorro C (2002). Different patterns of relapse after autologous peripheral blood stem cell transplantation in multiple myeloma: clinical results of 280 cases from the Spanish Registry. *Haematologica*.

[8] Zeiser R, Deschler B, Bertz H, Finke J, Engelhardt M (2004). Extramedullary vs medullary relapse after autologous or allogeneic hematopoietic stem cell transplantation (HSCT) in multiple myeloma (MM) and its correlation to clinical outcome. *Bone Marrow Transplantation*.

[9] Varettoni M, Corso A, Pica G, Mangiacavalli S, Pascutto C, Lazzarino M (2010). Incidence, presenting features and outcome of extramedullary disease in multiple myeloma: a longitudinal study on 1003 consecutive patients. *Annals of Oncology*.

[10] Patriarca F, Prosdocimo S, Tomadini V, Vasciaveo A, Bruno B, Fanin R (2005). Efficacy of bortezomib therapy for extramedullary relapse of myeloma after autologous and non-myeloabiatlve allogeneic transplantation. *Haematologica*.

[11] Paulus A, Swaika A, Miller KC (2011). Clinical relapse in a patient with multiple myeloma presenting as an atrial plasmacytoma. *Journal of Clinical Oncology*.

[12] Durie BG, Harousseau JL, Miguel JS (2006). International uniform response criteria for multiple myeloma. *Leukemia*.

[13] Durie BG, Harousseau JL, Miguel JS (2006). Erratum: international uniform response criteria for multiple myeloma. *Leukemia*.

[14] Durie BG, Harousseau JL, Miguel JS (2007). International uniform response criteria for multiple myeloma. *Leukemia*.

[15] Billecke L, Penas EM, May AM (2012). Similar incidences of TP53 deletions in extramedullary organ infiltrations, soft tissue and osteolyses of patients with multiple myeloma. *Anticancer Research*.

[16] Dispenzieri A, Kyle R, Merlini G (2009). International Myeloma Working Group guidelines for serum-free light chain analysis in multiple myeloma and related disorders. *Leukemia*.

[17] Kröger N, Asenova S, Gerritzen A, Bacher U, Zander A (2010). Questionable role of free light chain assay ratio to determine stringent complete remission in multiple myeloma patients. *Blood*.

[18] Pratt G, Mead GP, Godfrey KR (2006). The tumor kinetics of multiple myeloma following autologous stem cell transplantation as assessed by measuring serum-free light chains. *Leukemia and Lymphoma*.

[19] Dawson MA, Patil S, Spencer A (2007). Extramedullary relapse of multiple myeloma associated with a shift in secretion from intact immunoglobulin to light chains. *Haematologica*.

[20] Hobbs JAR, Drayson MT, Sharp K, Harding S, Bradwell AR, Mead GP (2010). Frequency of altered monoclonal protein production at relapse of multiple myeloma. *British Journal of Haematology*.

[21] Kühnemund A, Liebisch P, Bauchmüller K (2009). ’Light-chain escape-multiple myeloma’-an escape phenomenon from plateau phase: report of the largest patient series using LC-monitoring. *Journal of Cancer Research and Clinical Oncology*.

[22] Donato LJ, Zeldenrust SR, Murrat DL, Katzmann JA (2011). A 71-year old woman with multiple myeloma status after stem cell transplantation. *Clinical Chemistry*.

[23] Bradwell AR (2010). *Serum Free Light Chain Analysis (Plus Hevylite)*.

